# Supplementary material for: Structured connectivity in the output of the cerebellar cortex
Source: Nat Commun. 2024 Jul 9;15:5563. doi: 10.1038/s41467-024-49339-1 (PMC11233638; doi:10.1038/s41467-024-49339-1)
Supplement: Supplementary file 1 — Supplementary Information [file 41467_2024_49339_MOESM1_ESM.pdf]

Supplementary Material

Supplementary Figures 1-12 plus Supplementary Table

Structured connectivity in the output of the cerebellar cortex

Kim M. Gruver[1,2,3], Jenny W. Y. Jiao[1], Eviatar Fields[1,2], Sen Song[4], Per Jesper Sjöström[5], and Alanna J. Watt[1*]

[1] Department of Biology, McGill University, Montréal, QC, Canada.

[2] Integrated Program in Neuroscience, McGill University, Montréal, QC, Canada.

[3] Present address: Allen Institute for Brain Science, Seattle, WA, USA

[4] Laboratory of Brain and Intelligence and Department of Biomedical Engineering, Tsinghua University, Beijing, China.

[5] Department of Neurology and Neurosurgery, McGill University, Montréal, QC, Canada.

*Correspondence: alanna.watt@mcgill.ca

Supplementary Material

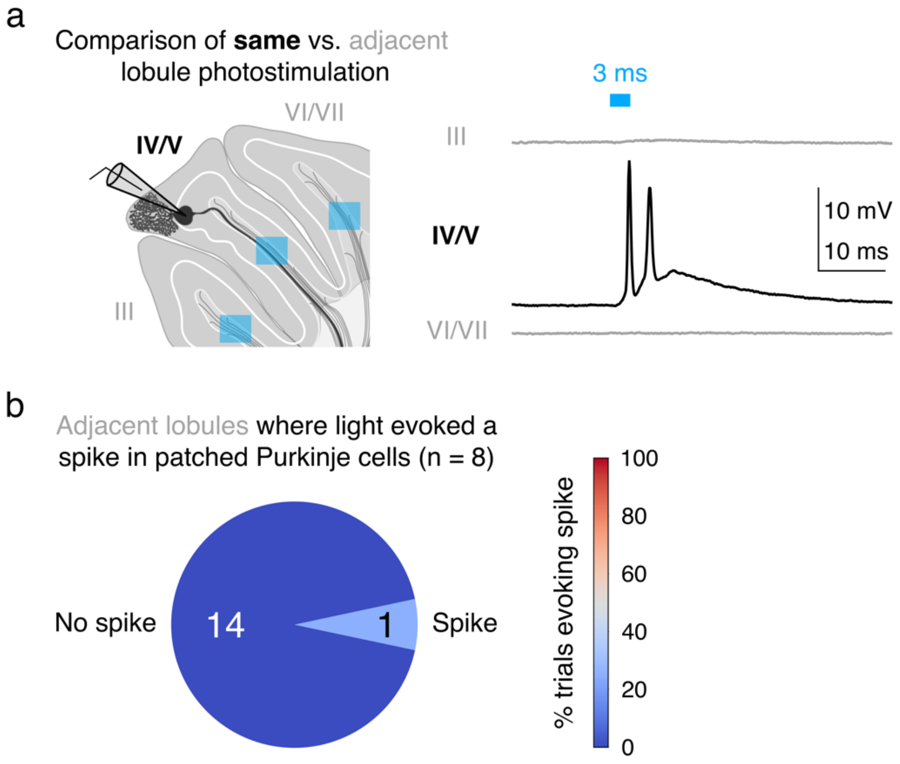

Supplementary Figure 1: Purkinje cells are rarely activated following photostimulation of adjacent lobules. **a**, Left, a cartoon showing the control stimulation paradigm to record antidromic Purkinje cell activation. Right, photostimulation of the lobule (IV/V) where the patched Purkinje cell resides ("same" lobule) elicits robust antidromic spiking, while photostimulation in each adjacent lobule (III and VI/VII) fails to elicit antidromic spiking. **b**, Pie chart showing the proportion of "adjacent" lobules tested where photostimulation unsuccessfully (14) vs. successfully (1) elicited an antidromic spike in recorded Purkinje cells (n = 15 neighbouring lobules tested, and 8 cells recorded). Colors indicate the percentage of trials where photostimulation of an adjacent lobule successfully evoked a spike (14 lobules with "no spike": 0 of all trials for 7 Purkinje cells, 1 lobule with "spike": 1 of 3 trials for a single Purkinje cell).

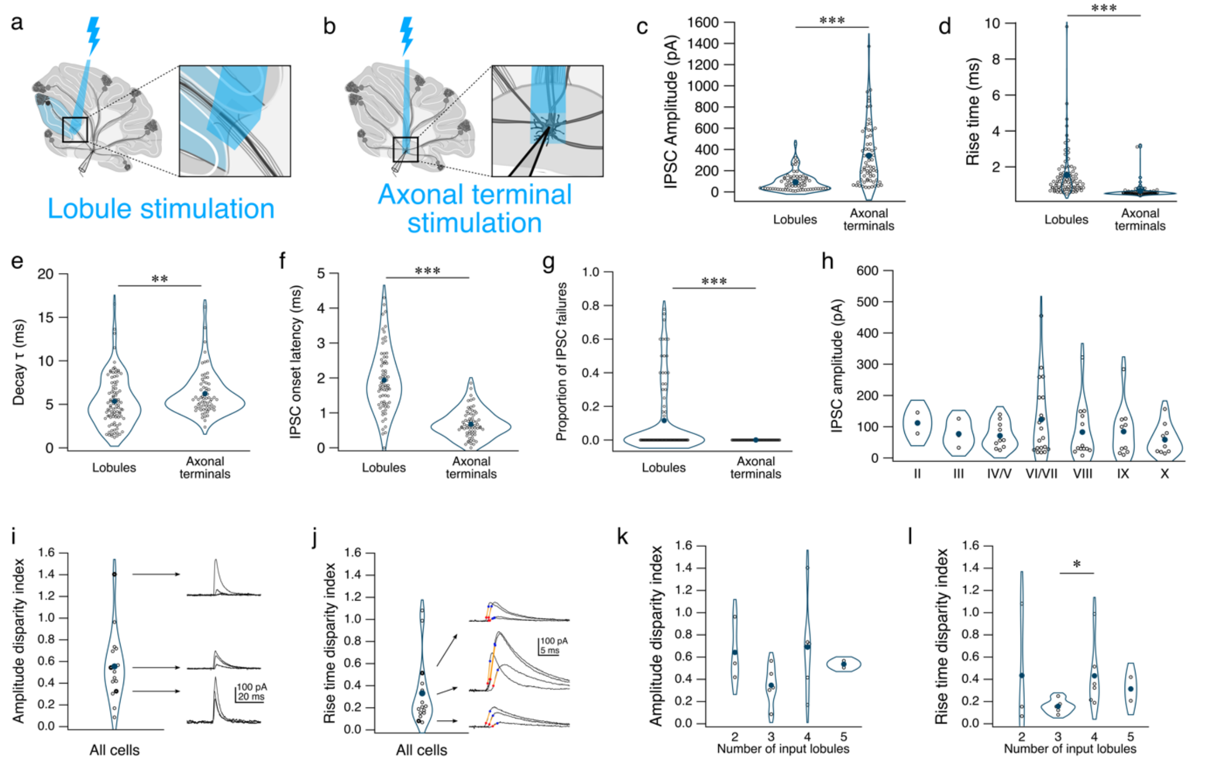

Supplementary Figure 2: Properties of inhibitory post-synaptic currents (IPSCs) following Purkinje cell stimulation. **a**, Cartoon showing area of photostimulation for "lobule stimulation," and **b**, "axonal terminal stimulation." **c-g**, IPSC amplitudes, rise times, weighted decay τ time constants, IPSC onset latency, and failures evoked following "lobule" or "axonal terminal" stimulation. **h**, IPSC amplitudes by lobule. **i-j**, Average IPSC amplitude disparity index for all cells with input from > 1 lobule: 0.55 ± 0.08, and average rise time disparity index for all cells with input from > 1 lobule: 0.33 ± 0.08. Traces to the right of each graph displaying examples of IPSCs with different disparity index values. **k-l**, Disparity indices for IPSC amplitudes and rise times by number of lobule inputs. *$P < 0.05$, **$P < 0.01$, ***$P < 0.001$. Unlabeled comparisons = not significantly different. Source data are provided as a Source Data file.

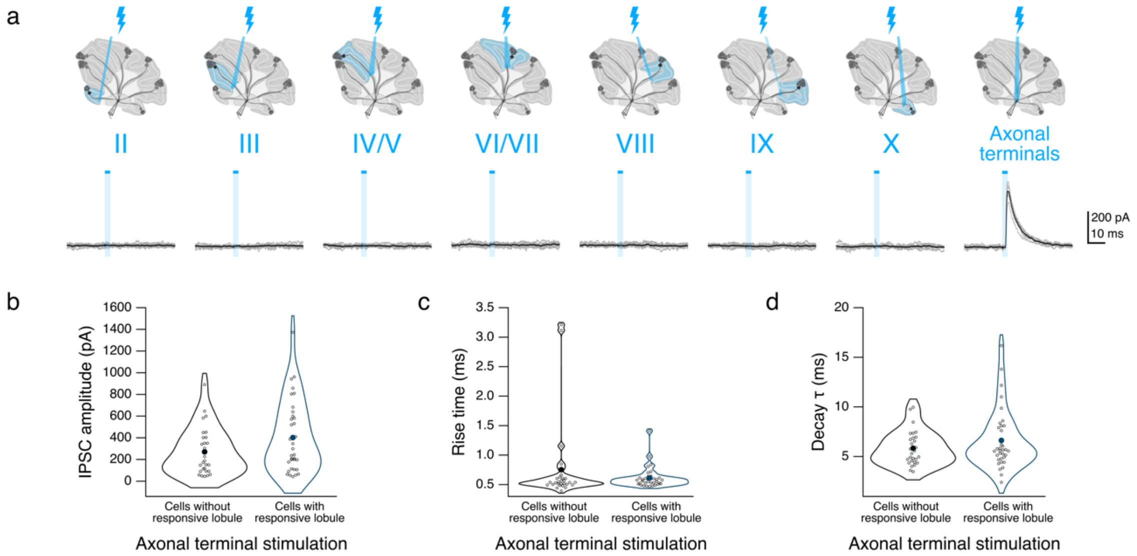

Supplementary Figure 3: Responses from cells with non-responsive lobules after lobule stimulation. **a**, Top, stimulation protocol as described in **Fig. 1b**. Bottom, example traces following lobule stimulation (left) and axonal terminal stimulation (right). **b**, Average IPSC amplitude for cells without responsive lobules versus cells with responsive lobules (Fig. 1) following axonal terminal stimulation. **c**, Rise times for cells without responsive lobules versus cells with responsive lobules following axonal terminal stimulation. **d**, Weighted decay τ time constants of IPSCs from cells without responsive lobules versus cells with responsive lobules. IPSC amplitudes, rise times, and decay τ time constants compared between cells with and without responsive lobules using non-parametric multiple comparison Mann-Whitney *U* test. Unlabeled comparisons = not significantly different. Source data are provided as a Source Data file.

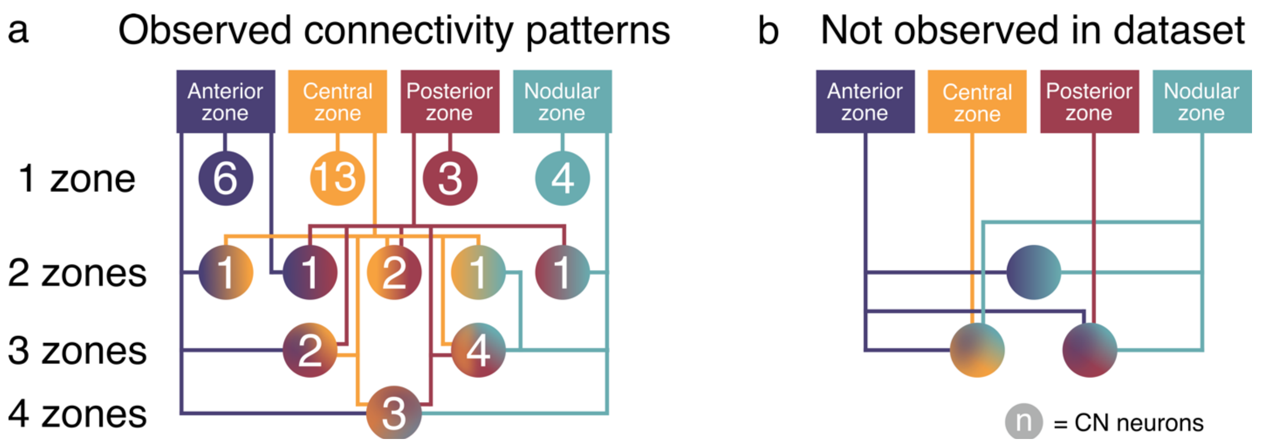

Supplementary Figure 4: **a**, Representation of observed Purkinje cell – CN neuron connectivity patterns based on cerebellar zones (circles). Numbers represent occurrences of each pattern in our dataset. **b**, The following connectivity patterns were not observed in our dataset: "AN," "ACN," and "APN" CN neurons.

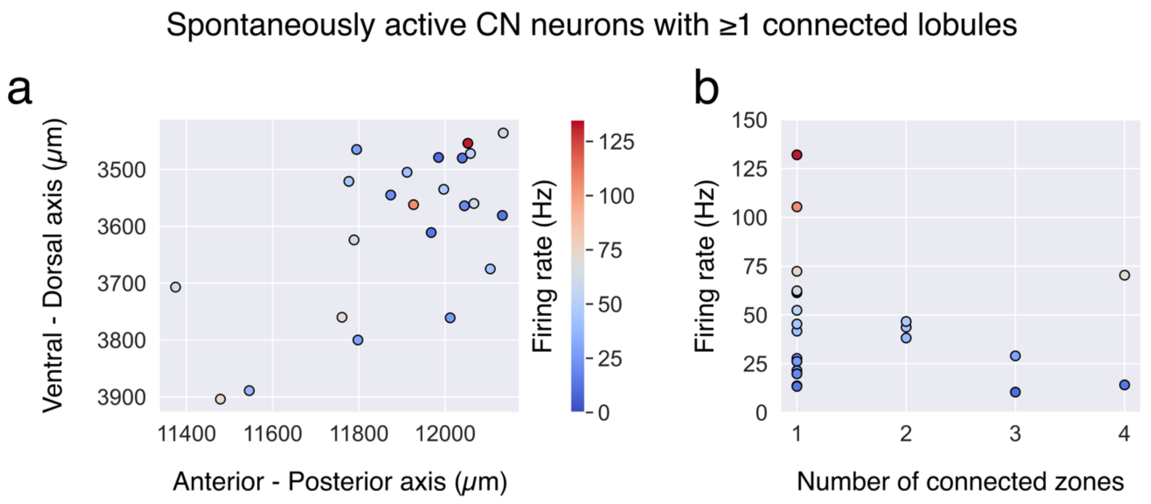

Supplementary Figure 5: Firing rates of spontaneously active CN neurons. **a**, Distribution of spontaneously active CN neurons with identified connections to Purkinje cells in ≥ 1 lobule in the ventral-dorsal and anterior-posterior (rostral-caudal) plane. **b**, Firing rates of CN neurons vs. number of connected zones. Source data are provided as a Source Data file.

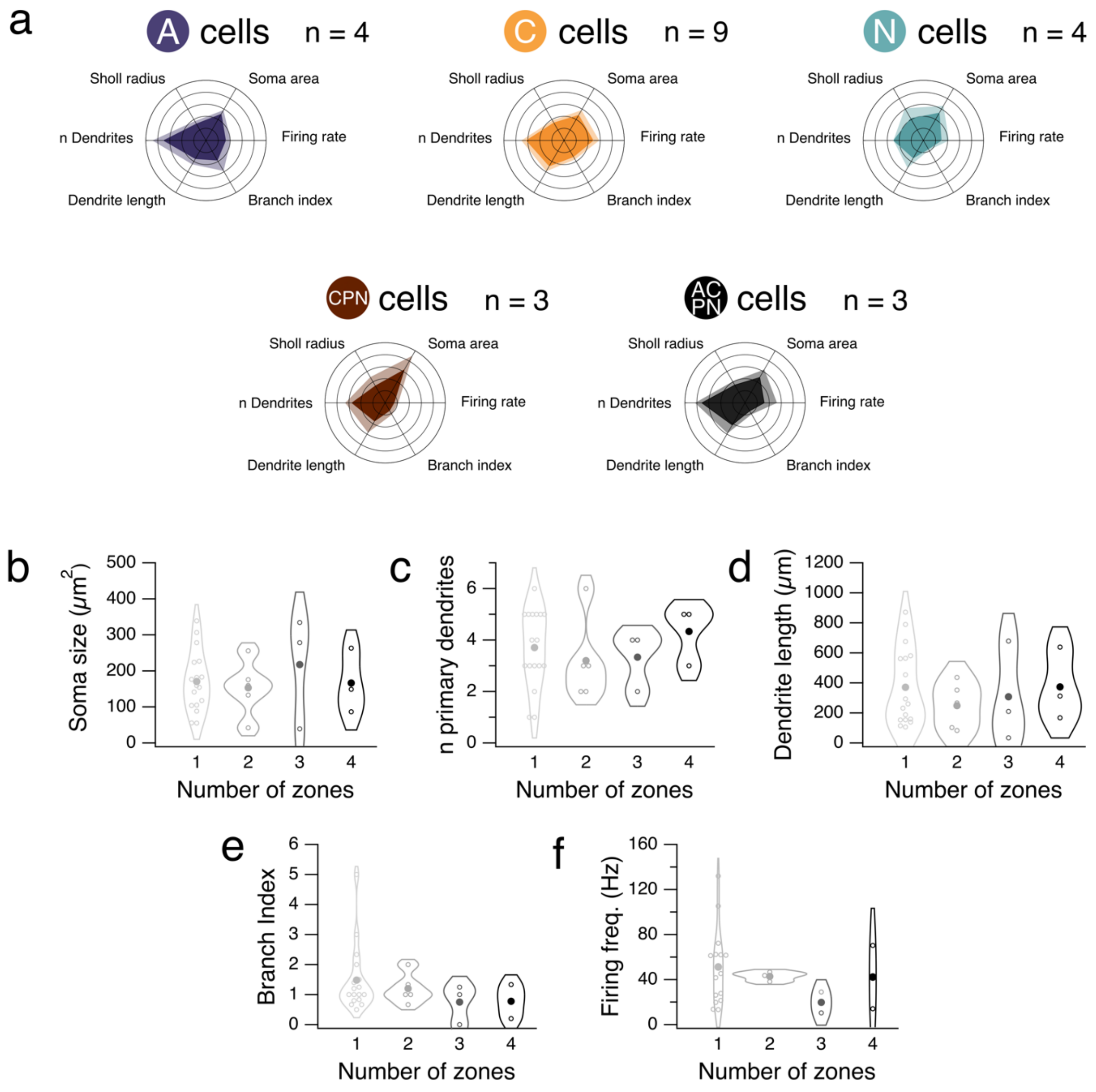

Supplementary Figure 6: Morphological and physiological properties of CN neurons. **a**, Polar plots displaying normalized average value of each measure per connectivity pattern for patterns with ≥ 3 cells per group. Shaded region indicates standard error. **b**, Soma size. **c**, Number of primary dendrites. **d**, Total dendrite length. **e**, Branch index at 25 μm. **f**, Firing frequency for cells by number of input zones. Unlabeled comparisons = not significantly different. Source data are provided as a Source Data file.

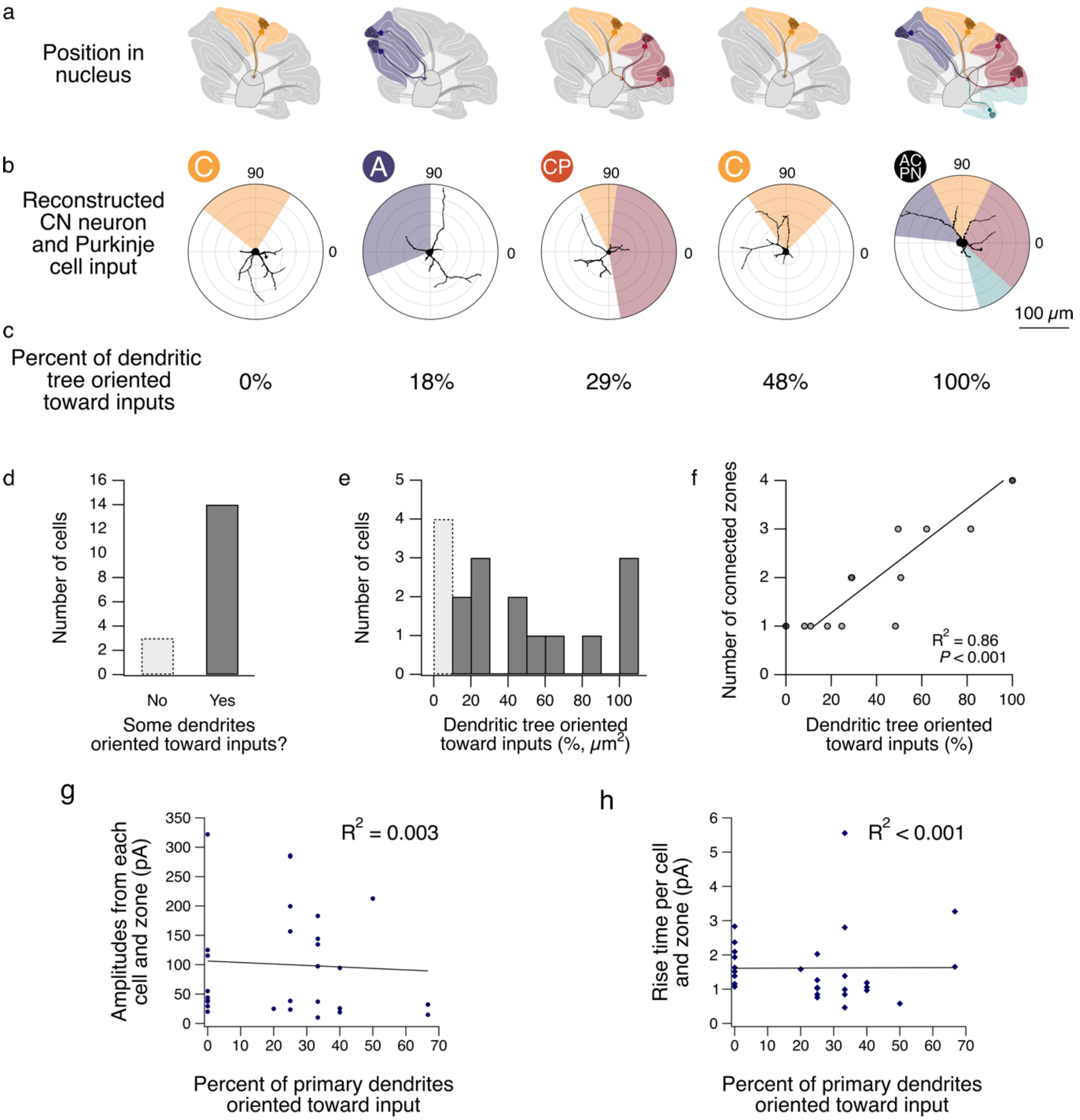

Supplementary Figure 7: CN dendrites are not preferentially oriented toward their zone inputs. **a**, Cartoons of cerebellar slices showing the relative position of individual CN neurons with different connectivity patterns. **b**, Polar plots showing the superimposed reconstructions of CN neurons from **a** with the input directions of the connected zones. **c**, Percent of dendritic tree for cells in **b** oriented toward their connected zones. **d**, Numbers of cells with and without dendrites oriented toward their inputs. **e**, Percent of CN neuron dendrite surface area oriented toward their

connected zones, and **f**, as a function of the number of connected zones. Number of connected zones vs. percent of dendritic tree oriented toward inputs, Pearson's r^2 correlation coefficient. **g**, Amplitudes for each input per cell vs. the number of primary dendrites oriented toward that input zone (e.g. a four-zone cell has four data points). **h**, Same as **g**, but the rise time for each input. Source data are provided as a Source Data file.

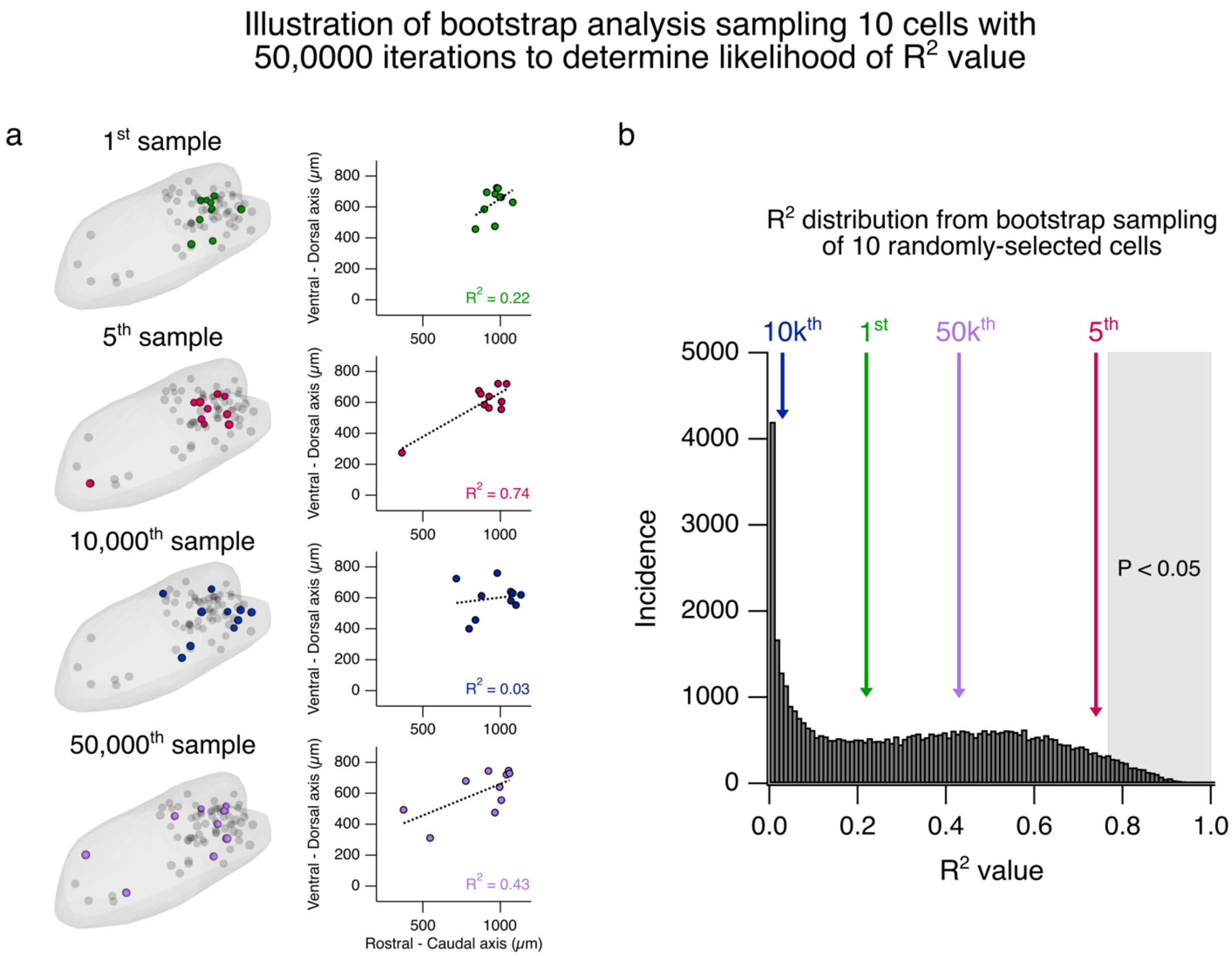

Supplementary Figure 8: Illustration of bootstrap analysis using 50,000 randomly selected samples of n cells to assess topographic organization within the CN. **a**, Left, renderings of CN in the sagittal plane depicting 10 randomly selected cells in the 1st, 5th, 10,000th, and 50,000th bootstrapped samples. Right, same as in **a, left,** but showing the plotted points for each CN neuron in the rostral-caudal and ventral-dorsal axes. Dotted lines and R^2 values in each graph represent the line of best fit for each sample of 10 cells. **b**, Distribution of R^2 values from the 50,000 bootstrapped samples. R^2 values from samples in **a** are highlighted with arrows. Shaded region depicts R^2 values that occurred in the top 5% of the distribution. Source data are provided as a Source Data file.

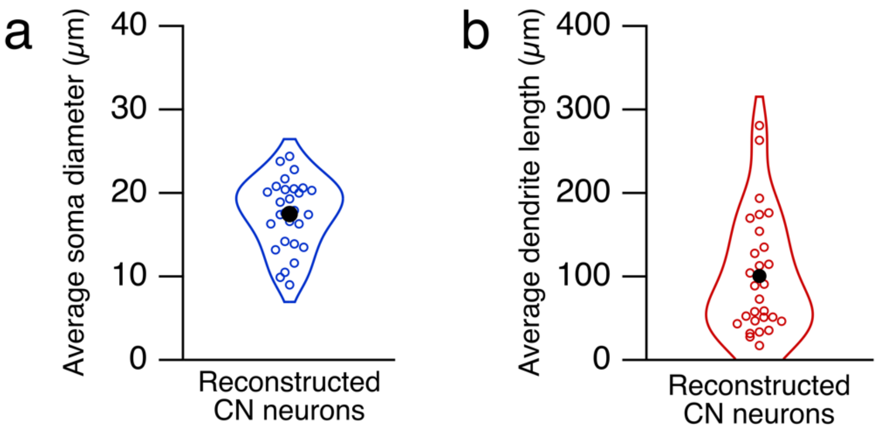

Supplementary Figure 9: Morphological properties from filled, reconstructed CN neurons. **a**, Average feret maximum soma diameter from Alexa-594-filled CN neurons. **b**, Average dendrite length from Alexa-594-filled CN neurons. n = 18 cells. Source data are provided as a Source Data file.

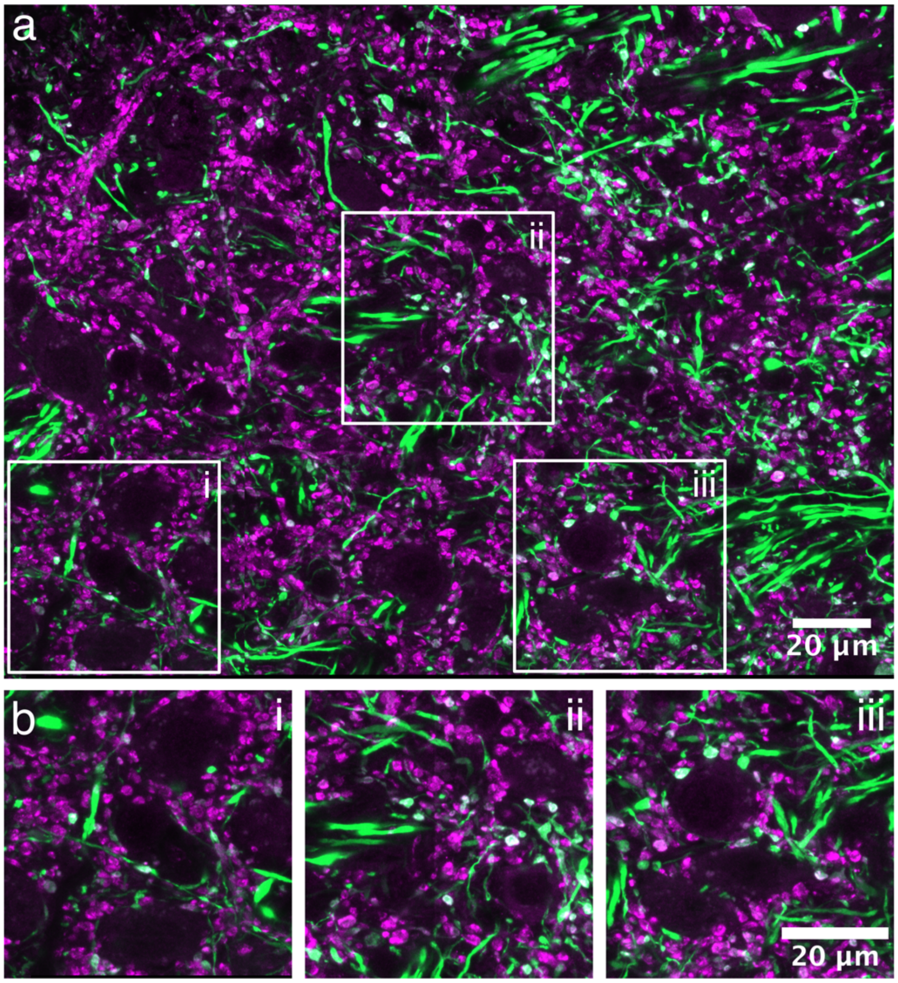

Supplementary Figure 10: Virally labeled Purkinje cell puncta colocalize with GABAergic presynaptic terminals. **a**, Representative image depicting labeling from intracranial injections of GFP in Purkinje cells (green) and puncta expressing vesicular GABA transporter (VGAT, magenta) in the fastigial nucleus. VGAT and GFP were colocalized in > 40% of GFP-positive puncta. **b**, Expansion of boxes i, ii, and iii shown in **a**.

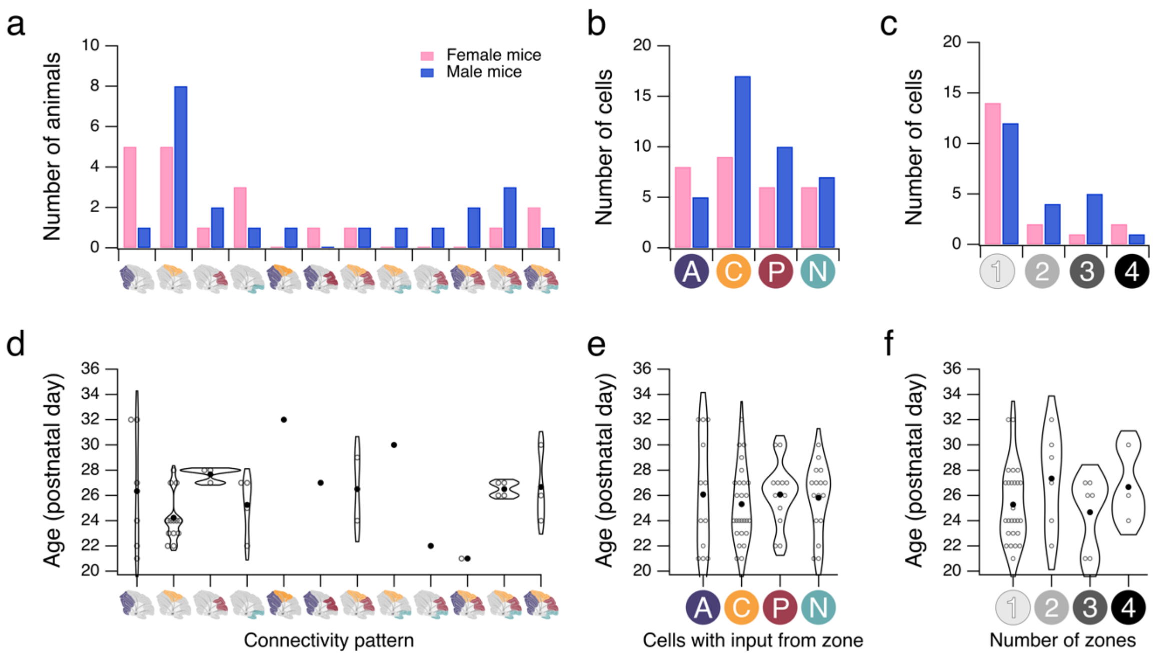

Supplementary Figure 11: No sex or age differences observed across different connectivity patterns. **a**, Distribution of observed connectivity patterns for female (pink) and male (blue) mice. **b**, Number of cells from female and male animals receiving input from each zone. **c**, Number of cells from female and male animals receiving input from n number of zones. **d**, Distribution of observed connectivity patterns across different ages. **e**, Distribution of different ages receiving input from each zone. **f**, Distribution of different ages receiving input from n number of zones. N = 22 female mice, 25 male mice. Unlabeled comparisons = not significantly different. Source data are provided as a Source Data file.

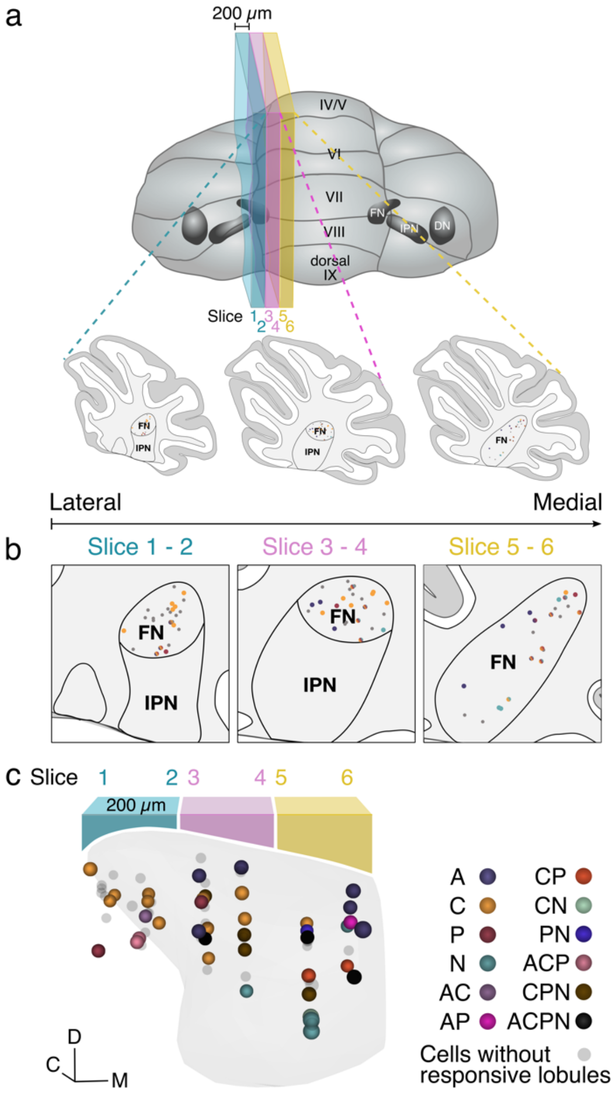

Supplementary Figure 12: A variety of Purkinje cell – CN neuron convergence is observed in each recorded slice. **a**, Cartoon depicting 3 continuous paravermis/vermis slices containing the fastigial CN. Slice orientations were recorded during each experiment to aid in mediolateral localization of patched CN neurons. **b**, Expansion of CN in **(a)** depicting the mediolateral localization of each patched CN neuron. For example, slice 1-2 refers to a single slice while describing which side was oriented facing up in the bath (1 = more lateral, 2 = more medial). **c**, 3-dimensional representation of patched CN neurons created using Brainrender software.

Animal	Number of positive pixels in channel 1	Number of positive pixels in channel 2	Number of colocalized positive pixels	Percent colocalized pixels/total positive pixels
Animal_ID1	52351	6756	139	0.24
Animal_ID2	75784	10140	88	0.10
Animal_ID3	5758	4844	41	0.39
Animal_ID4	107730	2808	157	0.14
Animal_ID5	35239	12014	140	0.30
Animal_ID6	8181	1392	0	0.0

Table S1: Colocalization of multiple viruses in virally labeled Purkinje cells. Number of positive pixels across imaging channels for six animals injected with fluorescently labeled viruses. Channels selected for colocalization analysis include the channel with the most viral expression (channel 1) and the channel with the second-most viral expression (channel 2). Numbers of positive pixels per channel represent the total number of positive pixels across >1 slices in imaging stack (2 μm interval).
