# Supplementary material for: Structured connectivity in the output of the cerebellar cortex
Source: Nat Commun. 2024 Jul 9;15:5563. doi: 10.1038/s41467-024-49339-1 (PMC11233638; doi:10.1038/s41467-024-49339-1)
Supplement: Supplementary file 3 — Reporting Summary [file 41467_2024_49339_MOESM3_ESM.pdf]

Corresponding author(s): Alanna Watt

Last updated by author(s): May 5, 2024

Reporting Summary

Nature Portfolio wishes to improve the reproducibility of the work that we publish. This form provides structure for consistency and transparency in reporting. For further information on Nature Portfolio policies, see our Editorial Policies and the Editorial Policy Checklist.

Statistics

For all statistical analyses, confirm that the following items are present in the figure legend, table legend, main text, or Methods section.

n/a	Confirmed	
☐	☒	The exact sample size (*n*) for each experimental group/condition, given as a discrete number and unit of measurement
☐	☒	A statement on whether measurements were taken from distinct samples or whether the same sample was measured repeatedly
☐	☒	The statistical test(s) used AND whether they are one- or two-sided<br>*Only common tests should be described solely by name; describe more complex techniques in the Methods section.*
☐	☒	A description of all covariates tested
☐	☒	A description of any assumptions or corrections, such as tests of normality and adjustment for multiple comparisons
☐	☒	A full description of the statistical parameters including central tendency (e.g. means) or other basic estimates (e.g. regression coefficient) AND variation (e.g. standard deviation) or associated estimates of uncertainty (e.g. confidence intervals)
☐	☒	For null hypothesis testing, the test statistic (e.g. *F*, *t*, *r*) with confidence intervals, effect sizes, degrees of freedom and *P* value noted<br>*Give P values as exact values whenever suitable.*
☒	☐	For Bayesian analysis, information on the choice of priors and Markov chain Monte Carlo settings
☐	☒	For hierarchical and complex designs, identification of the appropriate level for tests and full reporting of outcomes
☐	☒	Estimates of effect sizes (e.g. Cohen's *d*, Pearson's *r*), indicating how they were calculated

Our web collection on statistics for biologists contains articles on many of the points above.

Software and code

Policy information about availability of computer code

Data collection	Data acquisition was performed using custom IGOR Pro acquisition software. Two-photon imaging was acquired using ScanImage running in Matlab and images were used to create morphological reconstructions of CN neurons in Neurolucida. Confocal images were acquired using a LSM800 laser scanning confocal microscope at 10x and 20x with Zeiss Zen software.
Data analysis	Data analysis was largely performed using custom Igor Pro data analysis software that has been previously described. Clustering of lobules into transverse zones was performed using Seaborn's "clustergram" code based on Hamming distances using an unweighted average linkage in Python. Statistics were performed using JMP Pro 16 or Igor Pro 8 Wavemetrics software. Custom code was prepared based on the Brainrender package in GitHub to aid visualization of recorded cells (Fig. 4). Custom code was used to identify the nearest neighbor distances of virally-labeled puncta (Fig. 5). Custom code was used to extract the inter-spike interval duration from each trial to assess the ISI ratio (Fig. 6). Connectivity motifs were identified by comparing the actual vs. predicted probability of each combination by using a binomial distribution calculator available online: https://www.emathhelp.net/calculators/probability-statistics/binomial-distribution-calculator/<br>Custom code used to analyze the findings of this study and datasets analyzed in this study are available on Github: https://github.com/kgruver/StructuredConnectivity

For manuscripts utilizing custom algorithms or software that are central to the research but not yet described in published literature, software must be made available to editors and reviewers. We strongly encourage code deposition in a community repository (e.g. GitHub). See the Nature Portfolio guidelines for submitting code & software for further information.

Data

Policy information about availability of data

All manuscripts must include a data availability statement. This statement should provide the following information, where applicable:

- Accession codes, unique identifiers, or web links for publicly available datasets
- A description of any restrictions on data availability
- For clinical datasets or third party data, please ensure that the statement adheres to our policy

The authors declare that all data supporting the findings and custom code used to analyze the findings of this study are available on Github (https://github.com/kgruver/StructuredConnectivity). Source data are provided as a Source Data file.

Research involving human participants, their data, or biological material

Policy information about studies with human participants or human data. See also policy information about sex, gender (identity/presentation), and sexual orientation and race, ethnicity and racism.

Reporting on sex and gender	No human data or biological material were included in this study.
Reporting on race, ethnicity, or other socially relevant groupings	No human data or biological material were included in this study.
Population characteristics	No human data or biological material were included in this study.
Recruitment	No human data or biological material were included in this study.
Ethics oversight	N/A

Note that full information on the approval of the study protocol must also be provided in the manuscript.

Field-specific reporting

Please select the one below that is the best fit for your research. If you are not sure, read the appropriate sections before making your selection.

☒ Life sciences ☐ Behavioural & social sciences ☐ Ecological, evolutionary & environmental sciences

For a reference copy of the document with all sections, see nature.com/documents/nr-reporting-summary-flat.pdf

Life sciences study design

All studies must disclose on these points even when the disclosure is negative.

Sample size	Sample sizes were not predetermined. Since 16 different connectivity pattern combinations were possible, we collected data to adequately sample these patterns within the CN to avoid a sample-size bias toward over- or under-representing observation of connectivity patterns in our dataset.
Data exclusions	In electrophysiology experiments, cells were excluded from analysis if stimulation of each lobule was not sufficiently sampled (i.e. if recording was lost before assaying each region of interest). Recordings were also excluded if the input resistance value changed more than 20% of baseline. In intracranial viral labeling experiments, mice were excluded whose viral expression overlapped, or where only Purkinje cells from only one zone could be traced to the fastigial nucleus. Otherwise, if expression was non-overlapping between at least two zones for different fluorophores, the viral expression from those two fluorophores was sufficient to be included for analysis.
Replication	Data was repeated at least 3 times for different conditions. All attempts at replication were reported.
Randomization	Our study describes observational data rather than comparing different conditions. All cells were subjected to the same stimulation protocol, and cells were not assigned to different groups until after their input patterns were identified during each experiment. Cells were recorded from both female and male mice.
Blinding	Because the identity of each cell was unknown until after data analysis was performed, each experiment was performed blind.

Reporting for specific materials, systems and methods

We require information from authors about some types of materials, experimental systems and methods used in many studies. Here, indicate whether each material, system or method listed is relevant to your study. If you are not sure if a list item applies to your research, read the appropriate section before selecting a response.

Materials & experimental systems

n/a	Involved in the study
☐	☒ Antibodies
☒	☐ Eukaryotic cell lines
☒	☐ Palaeontology and archaeology
☐	☒ Animals and other organisms
☒	☐ Clinical data
☒	☐ Dual use research of concern
☒	☐ Plants

Methods

n/a	Involved in the study
☒	☐ ChIP-seq
☒	☐ Flow cytometry
☒	☐ MRI-based neuroimaging

Antibodies

Antibodies used	Primary antibodies used were: guinea pig anti-NeuN (1:500, abN90, Millipore) and rabbit anti-GFP (1:500, ab3080, Millipore). Secondary antibodies used were: DyLight 405 anti-guinea pig (1:500, 106475003, Jackson Immunoresearch) and goat Alexa 594 anti-rabbit (1:1000, A11037, Life Technologies).
Validation	Antibodies were tested at a range of concentrations, and optimal concentrations were chosen based on our preliminary testing and the literature. Secondary-only controls were included in all studies. The anti-NeuN antibody is "Part of the ValidAb™ range of highly validated, data-rich antibodies are confirmed to be reactive in mouse tissue for immunocytochemistry". For the anti-GFP antibody, the manufacturer website claims "This Anti-Green Fluorescent Protein Antibody is validated for use in ELISA, IC, IH, WB for the detection of Green Fluorescent Protein" in vertebrates.

Animals and other research organisms

Policy information about studies involving animals; ARRIVE guidelines recommended for reporting animal research, and Sex and Gender in Research

Laboratory animals	We crossed mice hemizygous for Purkinje cell-specific Cre [strain B6.Cg-Tg(Pcp2-cre)3555Jdhu/J; stock number: 010536; PCP2-Cre] with mice with a loxP-flanked stop cassette upstream of Channel-rhodopsin-2(ChR2)/H134R fused with enhanced YFP [strain: B6;129S-Gt(ROSA)26Sortm32(CAG–COP4*H134R/EYFP)Hze/J; stock number 012569; Ai32] to produce hemizygous PCP2-Cre/Ai32 mice21 that express ChR2 in Purkinje cells, or ChR2(H134R)-EYFP mice. We also used PCP2-Cre-negative mice to perform in-vivo multi-viral labeling experiments. Mice were housed in rooms with 12/12 hour day/night cycle in rooms maintained between 18-24º C and 30-70% humidity.
Wild animals	Study did not involve wild animals.
Reporting on sex	Both female and male animals were used in our study, and were randomly selected at the time of the experiment. We found no differences in observed connectivity patterns for mice of either sex. We show this data further in Supplementary Fig. 9. N = 22 female mice, 25 male mice.
Field-collected samples	Study did not involve samples collected from the field.
Ethics oversight	All animal procedures were approved by the McGill Animal Care Committee, in accordance with guidelines established by the Canadian Council on Animal Care.

Note that full information on the approval of the study protocol must also be provided in the manuscript.

Plants

Seed stocks	*Report on the source of all seed stocks or other plant material used. If applicable, state the seed stock centre and catalogue number. If plant specimens were collected from the field, describe the collection location, date and sampling procedures.*
Novel plant genotypes	*Describe the methods by which all novel plant genotypes were produced. This includes those generated by transgenic approaches, gene editing, chemical/radiation-based mutagenesis and hybridization. For transgenic lines, describe the transformation method, the number of independent lines analyzed and the generation upon which experiments were performed. For gene-edited lines, describe the editor used, the endogenous sequence targeted for editing, the targeting guide RNA sequence (if applicable) and how the editor was applied.*
Authentication	*Describe any authentication procedures for each seed stock used or novel genotype generated. Describe any experiments used to assess the effect of a mutation and, where applicable, how potential secondary effects (e.g. second site T-DNA insertions, mosiacism, off-target gene editing) were examined.*
